# A New MRI-Based Model of Heart Function with Coupled Hemodynamics and Application to Normal and Diseased Canine Left Ventricles

**DOI:** 10.3389/fbioe.2015.00140

**Published:** 2015-09-23

**Authors:** Young Joon Choi, Jason Constantino, Vijay Vedula, Natalia Trayanova, Rajat Mittal

**Affiliations:** ^1^Department of Mechanical Engineering, Johns Hopkins University, Baltimore, MD, USA; ^2^Institute for Computational Medicine, Johns Hopkins University, Baltimore, MD, USA; ^3^Department of Biomedical Engineering, Johns Hopkins University, Baltimore, MD, USA

**Keywords:** full heart function, electromechanics, hemodynamics, heart failure, immersed boundary method

## Abstract

A methodology for the simulation of heart function that combines an MRI-based model of cardiac electromechanics (CE) with a Navier–Stokes-based hemodynamics model is presented. The CE model consists of two coupled components that simulate the electrical and the mechanical functions of the heart. Accurate representations of ventricular geometry and fiber orientations are constructed from the structural magnetic resonance and the diffusion tensor MR images, respectively. The deformation of the ventricle obtained from the electromechanical model serves as input to the hemodynamics model in this one-way coupled approach via imposed kinematic wall velocity boundary conditions and at the same time, governs the blood flow into and out of the ventricular volume. The time-dependent endocardial surfaces are registered using a diffeomorphic mapping algorithm, while the intraventricular blood flow patterns are simulated using a sharp-interface immersed boundary method-based flow solver. The utility of the combined heart-function model is demonstrated by comparing the hemodynamic characteristics of a normal canine heart beating in sinus rhythm against that of the dyssynchronously beating failing heart. We also discuss the potential of coupled CE and hemodynamics models for various clinical applications.

## Introduction

The heart is an organ that pumps blood around the body by repeated muscular contractions. There is a strong coupling between the motion of the heart wall and the blood flow (Sengupta et al., 2006; Pasipoularides, 2010). Despite extensive research over the last few decades to understand cardiac functioning in health and disease, a comprehensive understanding of the heart function, especially with regard to the effect of myocardial abnormalities on cardiac hemodynamics, is still lacking due to the complexity of the cardiac structure (Arts et al., 1992; Noble, 2002), electrophysiology (Nash and Hunter, 2000; Trayanova, 2011, 2014), wall mechanics (Guccione and McCulloch, 1993a; Guccione et al., 1993b; Holzapfel and Ogden, 2009) and the associated hemodynamics (Kilner et al., 2000; Pedrizzetti and Domenichini, 2005; Pasipoularides, 2010). Computational modeling provides a powerful modality for understanding heart function (Winslow et al., 2012; Henney et al., 2015), and while electrophysiology, mechanics, and hemodynamics are highly coupled in the functioning of the heart, much progress has been made in understanding cardiac function with “unimodal” computational models, i.e., models that are either physically detailed in the electrophysiology (Ashikaga et al., 2013; Rantner et al., 2013; Trayanova and Boyle, 2014; McDowell et al., 2015), the mechanics (Sheikh et al., 2012), or the hemodynamics (Seo and Mittal, 2013b; Seo et al., 2014; Vedula et al., 2015). Additionally, although a number of studies have combined high-resolution biophysics-based models of cardiac electrophysiology and mechanics (Gurev et al., 2011; Provost et al., 2011; Land et al., 2012; Lim et al., 2012, 2015; Howard et al., 2013; Hu et al., 2014) to create cardiac electromechanics (CE) models, the hemodynamics has been typically modeled using lumped-element models (LEMs) (Aguado-Sierra et al., 2011). While these LEMs can provide gross predictions of mean pressure and rate of work in the heart, they do not provide any information regarding intrachamber fluid flow. In the latter class of models, the hemodynamics inside the heart chambers is usually modeled in detail using the Navier–Stokes equations but the motion of the endocardium is prescribed (usually from cardiac imaging or via empirical function) and lacks any direct coupling with the electromechanics (Pedrizzetti and Domenichini, 2005; Schenkel et al., 2009; Le et al., 2012; Zheng et al., 2012).

Cardiac electromechanical (CE) modeling, linking electrophysiological and mechanics modeling, has made advances over the previous decade; however, it is only in the last few years that image-based modeling and patient-specific approaches to CE, such as simulation of resynchronization therapy for dyssynchronous heart failure (HF), have been developed (Niederer et al., 2011, 2012; Hu et al., 2013, 2014; Krishnamurthy et al., 2013; Lumens et al., 2013). Concomitantly, hemodynamics modeling approaches have begun to incorporate the realistic geometries of the heart, with the endocardial motion extracted from medical images (Doenst et al., 2009; Schenkel et al., 2009; Mihalef et al., 2011; Seo and Mittal, 2013b; Chnafa et al., 2014; Vedula et al., 2015). With the advent of these approaches, it now appears possible (Nordsletten et al., 2011) to combine image-based CE modeling with Navier–Stokes-based hemodynamics (NSH) simulations, i.e., to create a model that incorporates the three different physical domains (electrical, mechanics, and hemodynamics) in such a way that the output of the electromechanics simulations serves as input to the hemodynamics simulations, rather than the latter incorporating the endocardial motion directly from images.

There are in fact a number of applications in cardiac research where heart-function models that couple physically realistic models of electromechanics as well as hemodynamics, could significantly enhance our understanding of the associated mechanisms and phenomena of interest. Of particular interest in this context is the relationship between the heart-wall motion and blood flow under conditions of dyssynchronous HF (Breithardt et al., 2002; Leclercq and Hare, 2004; Gurev et al., 2011; Provost et al., 2011; Lumens et al., 2013). For instance, such models can shed light on the effect of dyssynchrony on the residence time of blood through the ventricle and in doing so, help stratify the risk of ventricular thrombogenesis. These models could also provide insights into the implications of dyssynchrony for mitral and aortic valve regurgitation. Models could garner detailed information on the intraventricular pressure gradients, with implications for pinpointing the localized remodeling of the ventricular myocardium and could also be used to calibrate low-order lumped-element models.

In addition to dyssynchronous HF, such high-fidelity coupled models of heart function could be employed in a large variety of other cardiac problems; these include the prediction of left ventricle thrombus formation in infarcted ventricles (Son et al., 2012), study of systolic anterior motion (SAM) in patients with acute hypertrophic cardiomyopathy (Jiang et al., 1987), optimization of left ventricular-assist device (Long et al., 2014; Callington et al., 2015) implantation in patients with compromised ventricles, regurgitation in natural and prosthetic mitral valves, assessment of the effect of atrial fibrillations on cardiac performance (Koizumi et al., 2015), and surgical planning for procedures, such as leaflet plication (McIntosh et al., 1992; Padala et al., 2009) and hypertrophic resection (Maron, 2002). Such models could also be used for complex surgical therapies, such as cardiomyoplasty (El Oakley and Jarvis, 1994), where skeletal muscle is wrapped around a failing ventricle and activated using a pacemaker to provide cardiac assist. Finally, changes in the electromechanical function and consequently, the hemodynamics arising from generic defects in the cellular contractile apparatus could be successfully investigated using such a multi-scale combined approach.

The development of methodologies to construct realistic and/or patient-specific models that incorporate physically detailed models of the electrophysiology, the mechanics as well as the hemodynamics are, however, currently lacking. The reason for this slower progress is that assembling a high-fidelity, coupled model that incorporates all three physical domains, remains a very challenging task. First, translating physiological information (fiber orientation, tissue properties, lumen geometry, etc.) into computational models remains highly challenging, and continues to be a process that requires significant human effort. Second, each of the electrical, mechanical, and hemodynamic domains involve complex non-linear equations, which are extremely difficult to solve even on their own. For instance, we estimate that a well-resolved, hemodynamic simulation of one cardiac cycle of flow in a human left ventricle by itself would require O(10^18^) floating-point operations. In such simulations, the time step is typically limited by the well-known Courant–Freidrichs–Lewey (CFL) stability constraint (Ferziger and Peric, 2012). However, coupling with electromechanics can introduce more stringent time-scales that can increase the overall computational cost. In addition, such coupled solvers are seldom monolithic, and while the solver for each physical domain could be optimized for high performance on modern parallel computers, the coupled solver might suffer from computational inefficiencies and overhead.

The objective of the current study is to describe a new methodology that couples an image-based, biophysically detailed, multi-scale CE model of the heart to a high-fidelity NSH model. Such a combined, heart-function model overcomes some of the limitations of previous modeling approaches in elucidating CE–fluidic interactions. The one-way coupled model developed here is computationally efficient in that it allows the electromechanical and hemodynamic models to be integrated with time steps that are most appropriate for each physical model, and this type of heart-function model is best suited for fundamental parametric investigations of the effect of myocardial abnormalities on cardiac hemodynamics. To the best of our knowledge, this model of cardiac function is new and we demonstrate the potential utility of this model by investigating the hemodynamics in the canine left ventricle and implications for blood transport and pressure in a normal as well as dyssynchronously failing canine heart models.

## Materials and Methods

### Cardiac electromechanics model

To achieve the objective of this study, we employed image-based electromechanical models of the normal and failing canine hearts, combining electrophysiological and mechanical behavior of the heart, which have been described recently (Vadakkumpadan et al., 2009, 2010a; Gurev et al., 2011; Provost et al., 2011; Trayanova, 2011; Trayanova et al., 2011; Hu et al., 2013, 2014). We note that the canine data were originally acquired over 10 years ago for a number of scientific purposes, with the studies being approved by the Animal Care and Use Committee of the National Heart, Lung, and Blood Institute (Helm et al., 2006). This dataset is also publicly available at http://gforge.icm.jhu.edu/gf/project/dtmri_data_sets/wiki/.

**Figure 1:**
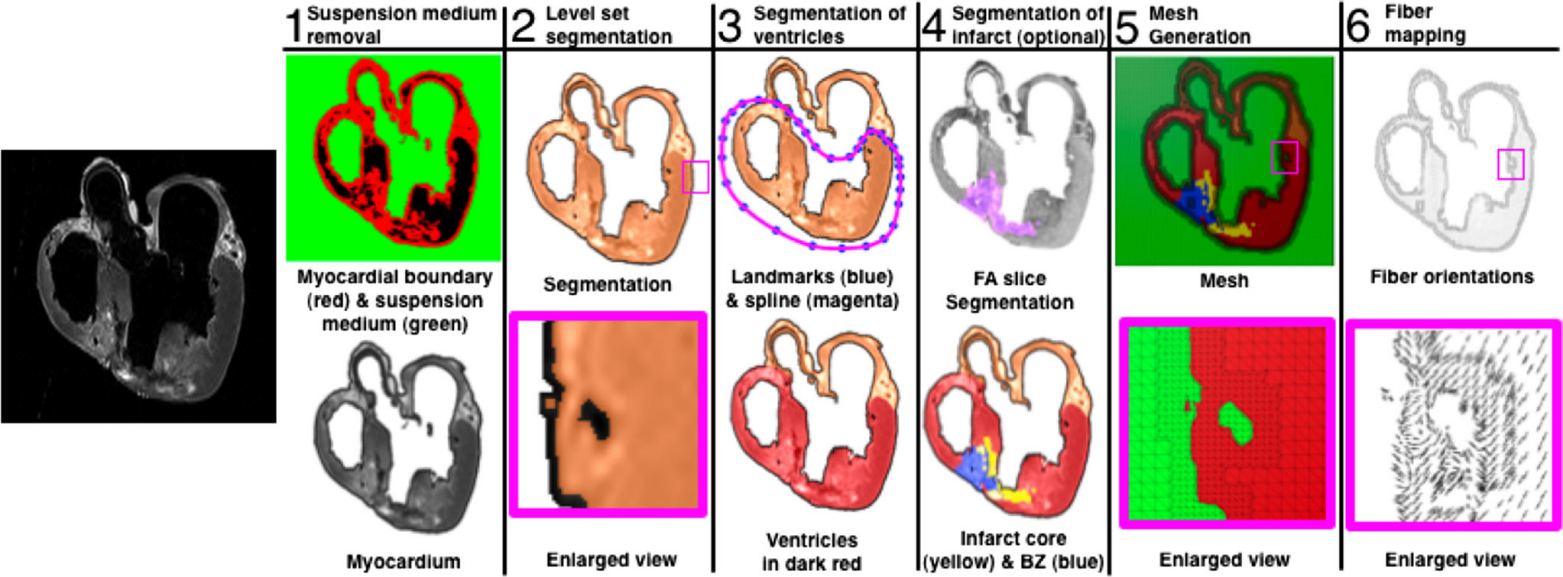
**Pipeline for MRI-based heart structure and geometry reconstruction**.

The ventricular geometries and the fiber/sheet architectures were generated from MR and diffusion tensor MR images, respectively. Figure 1 presents the generation of the model geometry from *ex vivo* MRI scans, the pipeline for which we have described previously (Vadakkumpadan et al., 2010b). Dilatation and ventricular wall thinning, as well as the changes in the fiber/sheet architecture, that are associated with HF were incorporated naturally into the electromechanical model of the HF canine ventricles, via the image-based geometry (Vadakkumpadan et al., 2009; Gurev et al., 2011).

**Figure 2:**
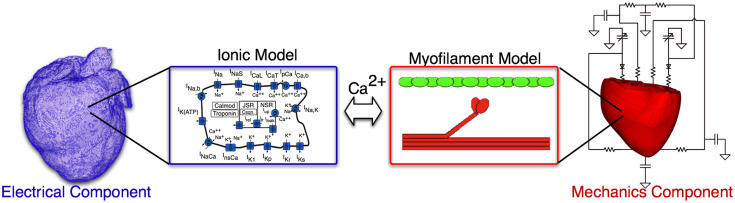
**Schematic of the electromechanical model**.

Briefly, the electromechanical model is composed of two parts: an electrical component and a mechanical component. The electrical component describes the propagation of electrical activity, which is governed by the mono-domain reaction-diffusion equations. The Luo–Rudy dynamic model (Luo and Rudy, 1994a) is employed to represent membrane dynamics; it is a generic action potential model that has been used in a number of previous electromechanical models (Luo and Rudy, 1994a,b; Kerckhoffs et al., 2006; Provost et al., 2011). The mechanical component describes the contraction of the heart and is based on the equations of continuum mechanics. The myocardium was assumed to be hyperelastic, nearly incompressible orthotropic material. The generation of active tension by the myocytes was represented by the Rice et al. (2008) model, which was parameterized for the canine heart by matching the data obtained from electromechanical wave imaging (an ultrasound experimental technique) as described earlier (Provost et al., 2011). A schematic of the electromechanical model is presented in Figure 2. A time step of 0.01 and 0.1 ms was used in the electrical and mechanical components of the model, respectively, as established in previous studies (Plank et al., 2008; Gurev et al., 2011). The parameters associated with cell-level modeling can be downloaded from the CellML website, https://www.cellml.org/.

The electrical and mechanics components were weakly coupled to minimize the computational effort; the membrane kinetics model was incorporated in both the electrical and mechanics components. The local electrical activation times (time of cellular depolarization), which were obtained using the electrical component, determined the instants at which the combined ionic and cardiac myofilament model in the mechanics component was stimulated. A justification of this approach and further detail can be found in Gurev et al. (2011).

Pressure and volume boundary conditions are imposed on the ventricles by coupling the electromechanical model to the closed-system, lumped parameter model of the systemic and pulmonic circulatory system described by Kerckhoffs et al. (2007). In this model, the systemic and pulmonary circulations were each characterized by two Windkessel compartments in series to represent the arterial and capillary blood and the venous blood circulation systems; the atrium dynamics is represented using time-varying elastance models (Kerckhoffs et al., 2007).

Similar to our previous studies (Gurev et al., 2011; Constantino et al., 2012, 2013), the following changes were incorporated into the HF electromechanical canine heart model to reflect the remodeling of the electromechanical properties associated with HF. First, the longitudinal and transverse electrical conductivities in the failing ventricles, reflecting tissue level electrical properties, as indicated in Figure 2, were reduced by 20% of the values used in the normal ventricles – the latter values based on canine data from Akar et al. (2004). To incorporate the deranged calcium handling in HF model at the cell level (ionic model, electrical component, see Figure 2), we reduced the peak amplitude of the calcium transient by 50% and increased the relaxation rate by 30% of the values used in the normal ventricular model (O’Rourke et al., 1999). Lastly, the passive stress scaling constant, *C*, in the strain-energy function (passive mechanical tissue properties, see Figure 2) was decreased by 50% (Usyk et al., 2000) to incorporate the reduced stiffness of myocardial tissue in failing hearts (Jaber et al., 2008).

**Figure 3:**
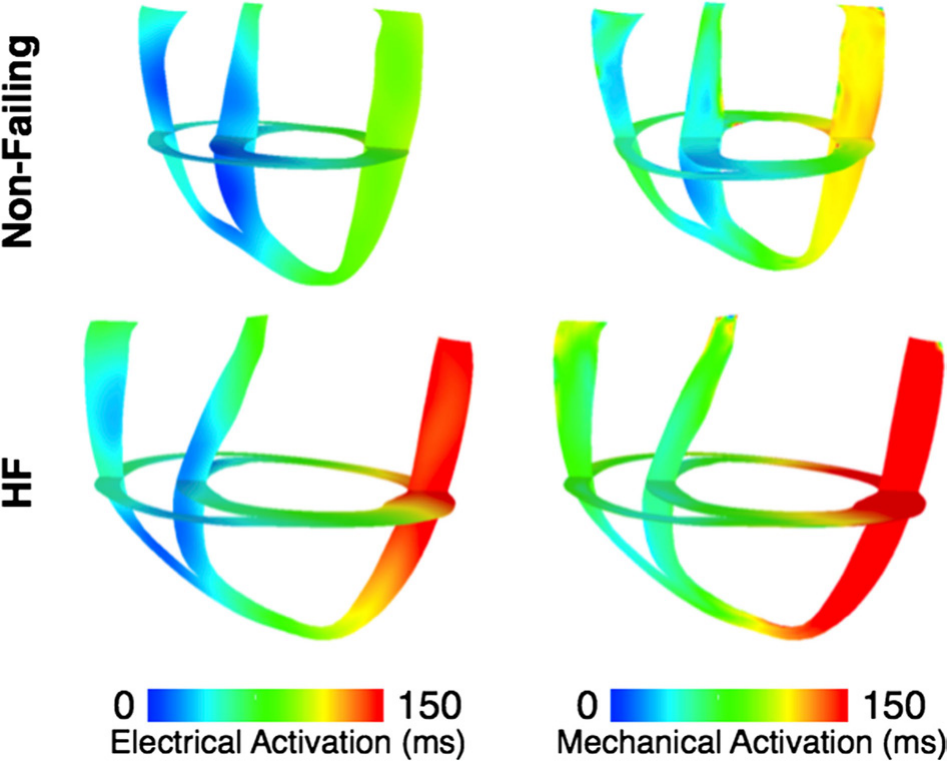
**Electrical activation times (left) and mechanical activation times (right) in the dyssynchronous non-failing (top) and dyssynchronous failing (bottom) canine ventricular models**.

To represent electrical activity in sinus rhythm (SR), the normal and failing ventricles were activated at discrete locations on the right ventricular (RV) and LV endocardium as if the activation originated from the Purkinje fibers, as demonstrated previously (Gurev et al., 2010, 2011; Constantino et al., 2012, 2013). The timing and locations of the stimuli were based on experimental 3D electrical propagation patterns (Durrer et al., 1970; Usyk et al., 2000; Ramanathan et al., 2006). Furthermore, two additional electromechanical models were generated, one normal and one failing, with a different SR activation sequence, i.e., left bundle branch block (LBBB), known to lead to dyssynchrony in contraction (Constantino et al., 2012, 2013). LBBB was modeled by stimulating the ventricles only at RV endocardial locations used in the normal SR simulations. An exhaustive description of all the electromechanics methodology used here, including image-based model development, mesh generation, fiber orientation assignment, representation of the electrical and mechanical properties of the myocardium, and numerical methods can be found in the detailed modeling methods paper by Gurev et al. (2011). Electrical and mechanical activation in the two LBBB models is presented in Figure 3, as an illustration of an output from the normal and failing electromechanical models. The characteristics of the four electromechanical models, two in SR, and two in LBBB activation used in this study, are presented in Table 1. Validation of the electromechanical models with experimental data can be found in a recent publication (Constantino et al., 2013).

**Table 1:** **Characteristics of the four canine heart models used in the simulations**.

	EDV (mL)	ESV (mL)	SV (mL)	EF (%)
Normal heart	41.2	23.4	17.8	43.2
SR activation
Normal heart	44.1	27.8	16.3	37.0
LBBB activation
Failing heart	116.4	100.2	16.2	13.9
SR activation
Failing heart	128.1	114.5	13.6	10.6
LBBB activation

*SR, sinus rhythm; LBBB, left bundle branch block; EDV, end-diastolic volume, ESV, end-systolic volume, SV, stroke volume, EF, ejection fraction*.

### Coupling of the electromechanics model to the hemodynamics model

The electromechanical model is coupled to the hemodynamics model by prescribing the motion of the endocardial surface during normal and dyssynchronous contraction obtained from the electromechanical model as a kinematic boundary condition to the flow simulation. Thus, the current approach is a one-way-coupled approach. Furthermore, since the two primary computational elements, the electromechanical solver and the hemodynamic solver, employ different numerical approaches and grid topologies, a procedure is needed to take the output of the LV kinematics generated by the electromechanical solver and prepare it for use in the hemodynamic solver. In particular, two issues are faced in this regard: first is the need for additional features, such as the left atrium (LA) and aorta (Ao), for the hemodynamic modeling, that are absent in the electromechanical model and second, is the inherent mismatch in the grid resolution and element topology employed by the two. We address both these issues by a two-step procedure: first, we generate a template surface model of the LV that has the additional anatomical features for the hemodynamic modeling and also has the appropriate element topology and grid resolution. This is followed by a template-based mapping procedure that registers the template to the individual time-frames from the electromechanical model, thereby ensuring the conformity of the mesh for the hemodynamic simulations.

**Figure 4:**
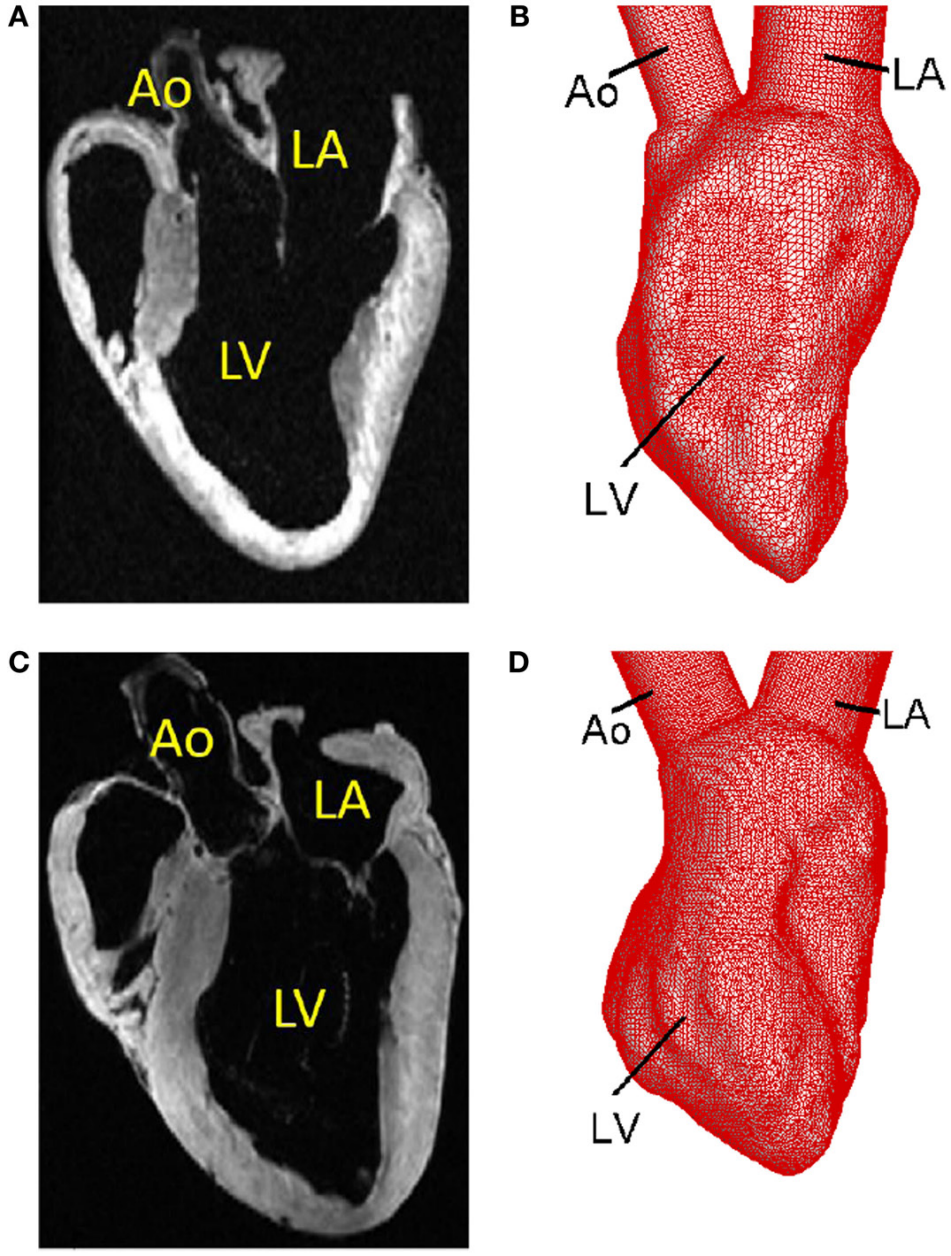
**Template surfaces generated from MR images at the unloaded state**. **(A)** MR image of the normal canine heart. **(B)** Triangulated template surface of the normal heart. **(C)** MR image of the failing canine heart. **(D)** Triangulated template surface of the failing heart.

As mentioned above, the MRI-based electromechanical models described above consist of the left and right ventricles and exclude the atria, Ao and pulmonary arteries. However, since we are mainly interested in the blood flow patterns in the LV, for cardiac flow simulation, we need features, such as the LA and Ao, in order to simulate the blood flow into and out of the ventricle. To overcome this issue, we first create a template of the LV based on the MR image of the heart at the unloaded state and add to it simplified geometric representations of LA and Ao (Figure 4); For the segmentation step, the images are subjected to Gaussian filtering for noise reduction and a mask is extracted out of the images by thresholding the image intensities using Seg3D^1^ image processing software. A region growing-based segmentation is then performed on the mask using Mimics (*Materialise Inc*.^2^) to extract the ventricular lumen and a surface mesh is generated using the surface wrap tool and CAD module of Mimics (*Materialise Inc*.) software suite. While the electromechanical model employs hexahedral elements, the template lumen surface is represented with a mesh consisting of triangular elements that is compatible with the requirements of the hemodynamic solver (Mittal et al., 2008). Furthermore, a high mesh density with elements ranging from about 24,000 to 48,000 is employed so as to satisfy the resolution requirements of the hemodynamic solver.

**Figure 5:**
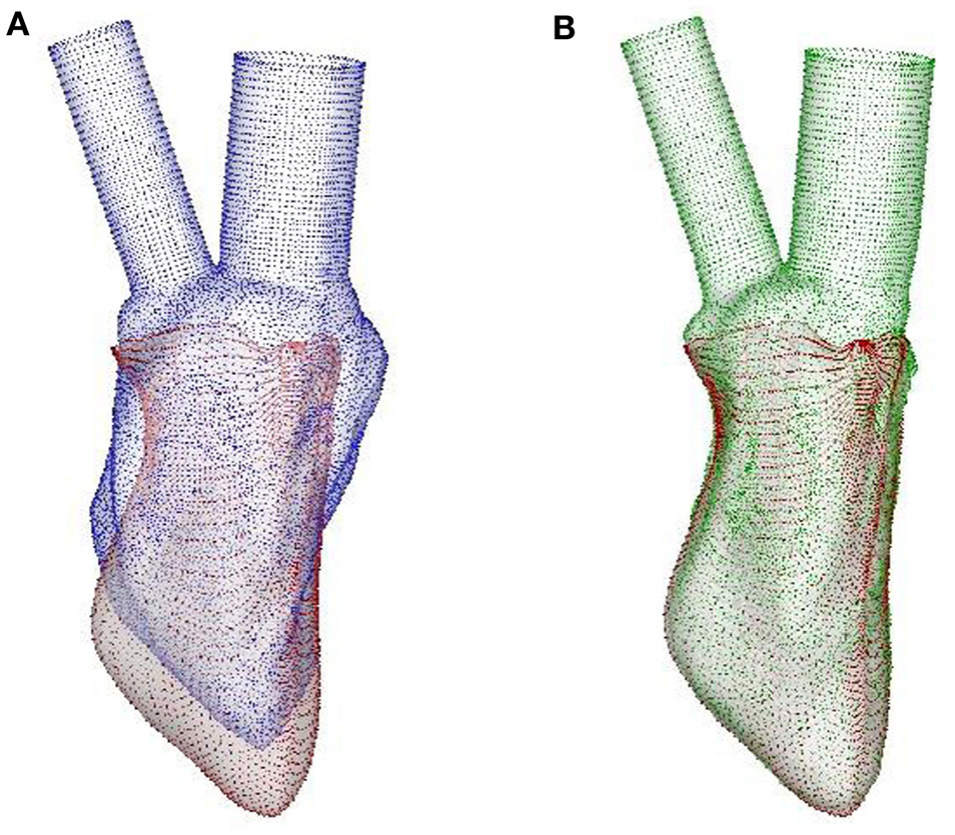
**LDDMM mapping of the template of normal canine LV endocardium to the target at SR**. **(A)** The template (blue surface) and the target (red surface), the latter being the output of the image-based CE model. **(B)** LDDMM-transformed template (green surface) matches the target reasonably well. LDDMM, large deformation diffeomorphic metric mapping; LV, left ventricle; SR, sinus rhythm; CE, cardiac electromechanics.

The ventricular surface of the template generated as described above is then deformed to match the LV endocardium at various stages in the cycle as obtained from the electromechanical simulation for each of the four cases investigated here. To accomplish this, the faces of each hexahedral elements of the mechanical mesh are first subdivided into fifty triangular elements to generate the ventricular luminal surface for the hemodynamic simulator. Then, the template LV surface is mapped to the “target” LV endocardium obtained from the electromechanical model using a diffeomorphic mapping algorithm known as Large Deformation Diffeomorphic Metric Mapping method (LDDMM) (Glaunes et al., 2004; Younes, 2010) as shown in Figure 5. This mapping procedure ensures that the surface grid is conformal (i.e., has the same number and connectivity of elements) from one time-frame to another.

The LDDMM mapping of the template (blue surface) to the target (red surface) of the normal canine ventricle with sinus activation is shown in Figure 5A at the beginning of diastole. The green surface in Figure 5B is the template which is LDDMM mapped to the target (red surface), and it can be seen that the mapping matches the target reasonably well. The LDDMM template to target mapping was repeated for every key frame of the cardiac cycle, and the resulting mapped surfaces were used in the cardiac flow simulations described in the following section. The normal heart template (Figure 4B) was used to match both normal heart targets – i.e., with SR and LBBB activation. Similarly, the failing heart template (Figure 4D) was used for mapping both failing heart targets – SR and LBBB activation.

We note that no models of the mitral or aortic valve leaflets are included in this version of the ventricular model. The mitral valve in particular is expected to significantly affect the diastolic flow patterns in the ventricle (Seo et al., 2014); however, we do not currently have the information needed to correctly parameterize either the kinematics or the biomechanics of the canine mitral valve. The effect of excluding the mitral valve from a ventricular flow simulation is described in a separate study (Seo et al., 2014).

### Cardiac hemodynamics simulation using immersed boundary method

Once a kinematic heart model was constructed using LDDMM surface matching procedure as described above, it is then immersed into a non-body conformal Cartesian volume grid and a flow simulation was performed by solving the incompressible Navier–Stokes equations as:

(1) $$\rho \left[\frac{\partial u}{\partial t}+\left(u\cdot \nabla u\right)\right]=-\nabla p+\mu \nabla^{2}u$$

(2) ∇⋅u=0

where ***u*** is the velocity vector, ***p*** is the pressure, and ρ and μ are the density and the viscosity of the blood, respectively using the immersed boundary method (Mittal and Iaccarino, 2005; Mittal et al., 2008). The above governing equations are advanced in time by a second-order accurate projection method (Chorin, 1968; Zang et al., 1994) and the spatial discretization is also strictly second-order. The non-linear advection terms are discretized using the second-order Adam’s–Bashforth scheme, while the diffusion term is discretized using the second-order Crank–Nicolson method. The pressure Poisson equation, which is the most CPU intensive component of the solution process, is solved using a geometric multi-grid method (Mittal et al., 2008).

The Dirichlet velocity boundary conditions on the endocardial surface are imposed by using a multi-dimensional ghost-cell method (Mittal et al., 2008; Seo and Mittal, 2011) and these wall velocities are computed by interpolating the position coordinates of LDDMM-mapped heart models in time and later taking their temporal derivatives. The flow solver has been validated in a number of studies for flows past complex moving bodies (Dong et al., 2010; Zheng et al., 2010, 2013) and more recently, for a laboratory model of the LV (Vedula et al., 2014). Other application of this solver to cardiac flows can be found in Seo and Mittal (2013b), Seo et al. (2014), Choi et al. (2014), and Vedula et al. (2014, 2015).

The flow simulations for the four canine heart models were performed on a high-resolution grid (128 × 128 × 256) with the density and viscosity of blood assumed to be 1.04 g/cm^3^ and 0.035 g/cm s, respectively. The cardiac cycle duration for these canine heart models is 0.5 s and we have chosen a time step, Δ*t* = 10^−4^ s for all the flow simulations that corresponds to 5,000 time steps per cardiac cycle. Similar grids and time steps have been used in a number of previous LV simulations (Zheng et al., 2012; Seo and Mittal, 2013b; Seo et al., 2013a, 2014; Choi et al., 2014; Vedula et al., 2015) and found to be adequate for predicting the details of the flow in the LV.

### Methods for the analysis of intraventricular blood flows

The intraventricular blood flow patterns were visualized using vortex structures identified by the isosurfaces of swirl strength (Jeong and Hussain, 1995), which is defined as the second invariant of the velocity gradient tensor, and provides a frame-invariant measure of the strength of the rotational motions in the flow. We also compare the velocity vectors on the mid-plane of the ventricle between all the study cases and compare the inflow velocity magnitude at the center of the mitral annulus as a function of time.

We used Lagrangian particle tracking method to investigate blood transport in the LV (Seo et al., 2013a). The virtual particles (12,000 particle in this study) representing point-mass blood cells were randomly distributed throughout the entire computational domain (LA, LV, and Ao). The motion of the particles is governed by the kinematic equation, *dx*_0_/*dt* = *u*(x_0_) where, *x*_0_ is the position of a particle and *u*(*x*_0_) is the velocity of the fluid at the position of the particle obtained by solving the Navier–Stokes equations. The equation is integrated using a fourth-order Runge–Kutta (RK4) time integration method to obtain particle positions. Particles are continuously injected through the mitral inlet during diastole, and ejected through the Ao during systole are removed from the computational domain.

Following the particle tracking analysis performed previously (Bolger et al., 2007; Carlhall and Bolger, 2010; Hendabadi et al., 2013; Seo and Mittal, 2013b; Seo et al., 2013a), the ventricular volume is divided into four sub volumes depending on its constituents during a cardiac cycle: the inflow from the atrium is divided into direct inflow (I) that gets ejected in the same cycle, and retained inflow (II), which is composed of blood cells that are not ejected in the same cycle. Likewise, there is also the volume of blood in the ventricle that was present in the ventricle at the start of the cycle, which is ejected in the current cycle that is referred to as delayed ejection (III). Finally, there is the residual volume (IV) of blood that is present at the start of the cycle and stays in the ventricle at the end of the cycle.

In the present analysis, we focus on two metrics that characterize the transport of blood through the ventricle: direct ejection ratio (DR) [DR = I/(I + II)], which is defined as ratio of directly ejected inflow to the total inflow (which is also equal to the stroke volume); and washout ratio (WR) [WR = III/(III + IV)] defined as the ratio of delayed ejection volume (not the total ejected volume) to the total ventricular blood at the beginning of the cycle (which is equal to the end-systolic volume). It is also useful to identify the difference between WR and ejection fraction [EF = (I + III)/(I + II + III + IV)]. The former quantifies the transport of ventricular blood alone before and after the cardiac cycle, while the latter determines the net ejection or stroke volume during a cardiac cycle.

Work done by the ventricle to pump blood across it is given by the area under the pressure–volume (*P*–*V*) loop during the cardiac cycle. We compare the stroke work between all the four study cases to understand the effect of dyssynchrony and HF on ventricular performance. The volume-averaged pressure is used to generate the *P*–*V* loop defined as, $p_{\mathrm{LV}}=\left(1/V_{\mathrm{LV}}\right)\int \mathrm{pdV}$ where *V_LV_* is the ventricular volume and *p* is the local pressure obtained by solving the Navier–Stokes equations. The pre-load and after-load conditions are estimated from the lumped parameter network used in the previously described electromechanics model to determine the absolute pressure in the ventricle.

## Results

In the previous section, we described the methodology that couples an image-based, biophysically detailed, multi-scale electromechanics model of the heart to a high-fidelity hemodynamics model. In this section, we demonstrate the utility of our combined electromechanics-hemodynamics model by investigating the flow dynamics as well as the transport of blood cells in canine LVs under four different physiological conditions, i.e., through the LV in the normal and in the dyssynchronously failing canine hearts, each beating under SR and after LBBB activation.

### Diastolic flow patterns

**Figure 6:**
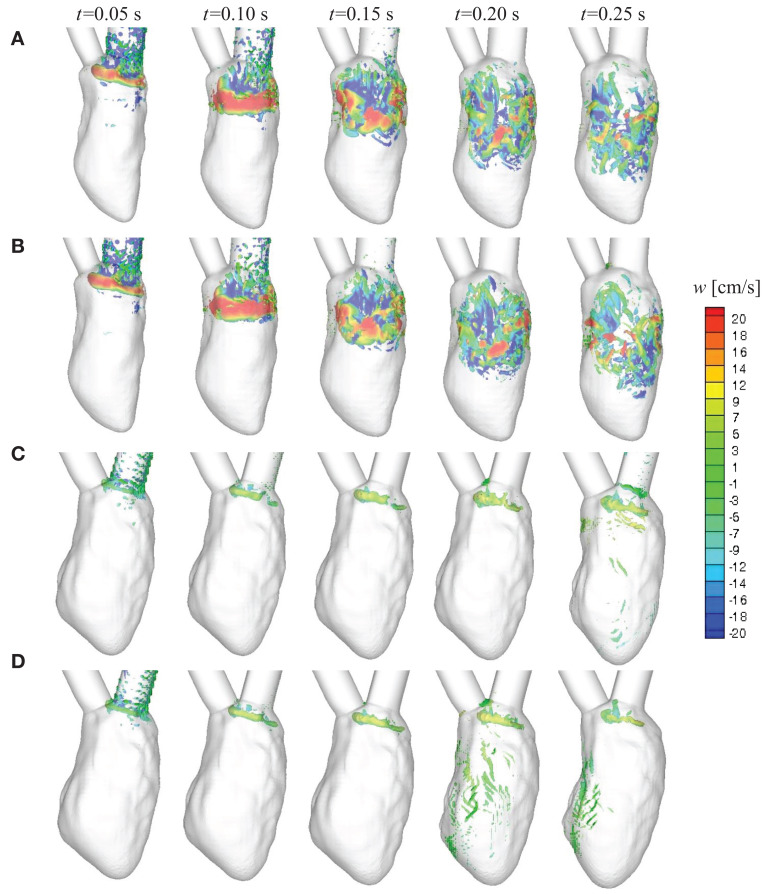
**Time-evolution of intraventricular vortex structures for the four canine heart models**. Vortex structures are identified by the swirl strength criterion (Jeong and Hussain, 1995) and are colored by the vertical velocity component *w*. **(A)** Normal heart with SR activation; **(B)** normal heart with LBBB activation; **(C)** failing heart with SR activation; **(D)** failing heart with LBBB activation. SR, sinus rhythm; LBBB, left bundle branch block.

We first compare the time-dependent diastolic flow patterns and the vortex structures in the normal and failing LVs to qualitatively examine ventricular efficiency. It has been shown in previous studies that optimal vortex formation during diastole is a characteristic of healthy ventricles (Kilner et al., 2000; Pedrizzetti and Domenichini, 2005; Gharib et al., 2006; Watanabe et al., 2008; Pasipoularides, 2010; Sengupta et al., 2012; Charonko et al., 2013; Chnafa et al., 2014; Seo et al., 2014). The vortex structures at selected time instances during diastole are shown in Figure 6. The colored contours represent the vertical (or longitudinal) velocity component. For the normal hearts, under SR (Figure 6A) or LBBB activation (Figure 6B), a distinct vortex ring is generated at the mitral annulus around *t* = 0.05 s. The vortex ring is pinched-off from the mitral annulus and propagates through the LV (*t* = 0.10 s) until it becomes unstable and begins to break down (*t* = 0.15 s) (Seo et al., 2014) into smaller vortex structures that penetrate past the mid-ventricular level by end-diastole for the normal hearts (*t* = 0.20–0.25 s). In contrast, the vortex ring that is formed for the failing hearts (Figures 6C,D) is extremely weak as characterized by the volume occupied by the isosurface of the swirl strength; it remains near the mitral inlet during diastole and does not propagate into the bulk of the ventricle throughout the filling phase.

**Figure 7:**
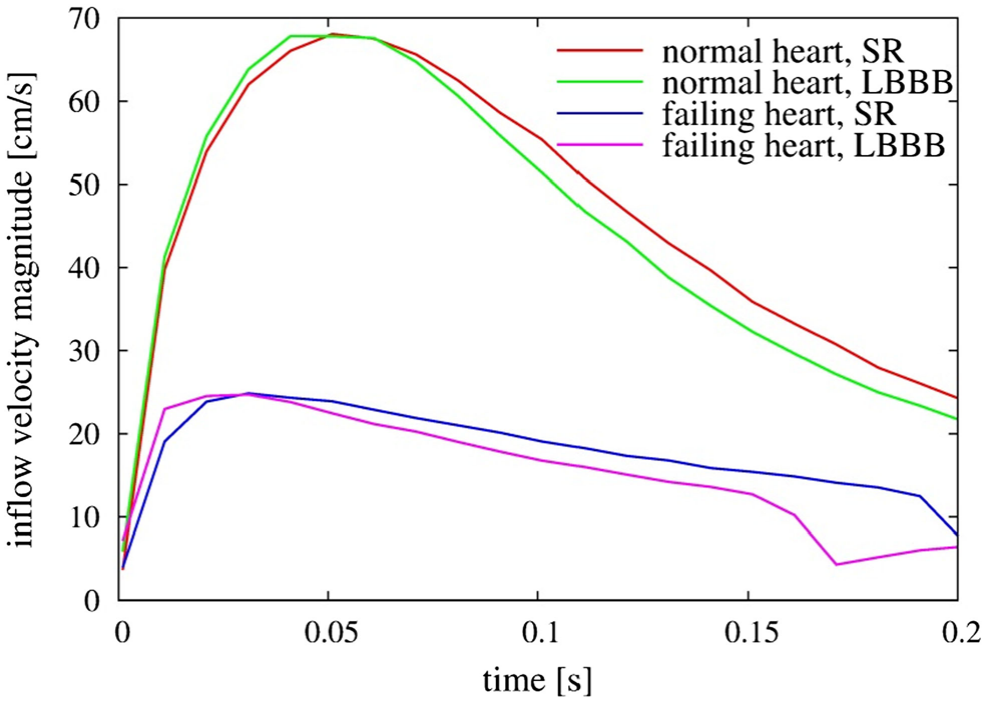
**Comparison of inflow velocity magnitude measured at the center of the mitral annulus as a function of time for the four heart models**. SR, sinus rhythm; LBBB, left bundle branch block.

**Figure 8:**
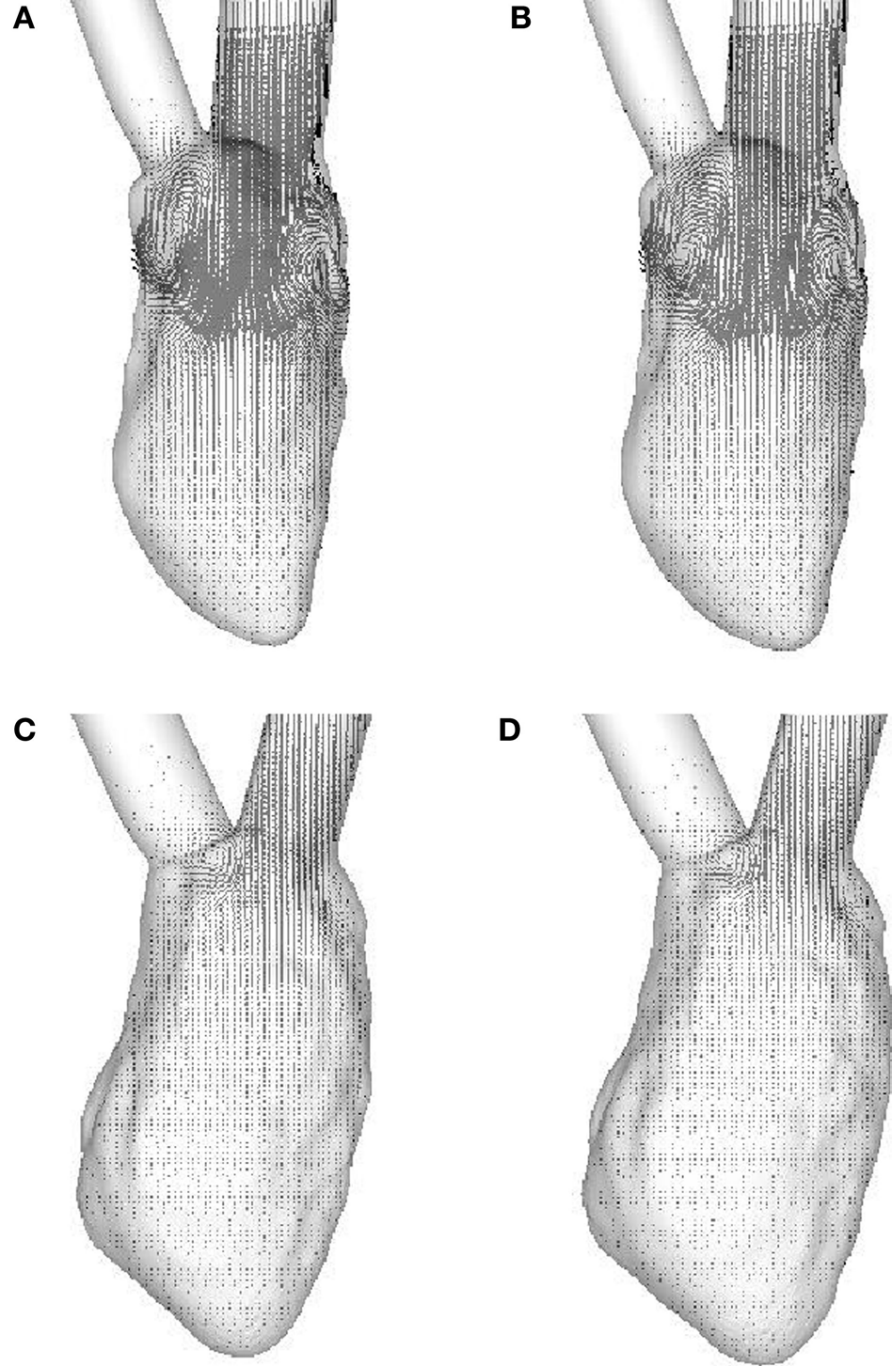
**Velocity vectors in the mid-plane of the ventricle at *t* **=** 0.10 s**. **(A)** Normal heart with SR activation; **(B)** normal heart with LBBB activation; **(C)** failing heart with SR activation; **(D)** failing heart with LBBB activation. SR, sinus rhythm; LBBB, left bundle branch block.

The weak vortex structures in the failing hearts are associated with the low magnitude of mitral inflow velocity, which results from the low ejection fraction. Figure 7 compares the velocity magnitude at the center of the mitral annulus for the four canine hearts considered in the present study. The inflow velocities in the failing hearts are much smaller than those in the normal hearts. The peak inflow velocity for LBBB activation appears a little earlier than that for the sinus activation, both in normal and in failing hearts. The intraventricular flow patterns are also visualized by the velocity vectors on the mid-plane of the ventricle (Figure 8) at *t* = 0.10 s. Similar to the vortex structures in Figure 6, the velocity field in Figure 8 also reveals weak vortex patterns associated with low blood flow velocity in the failing hearts.

### Blood transport in LV

**Figure 9:**
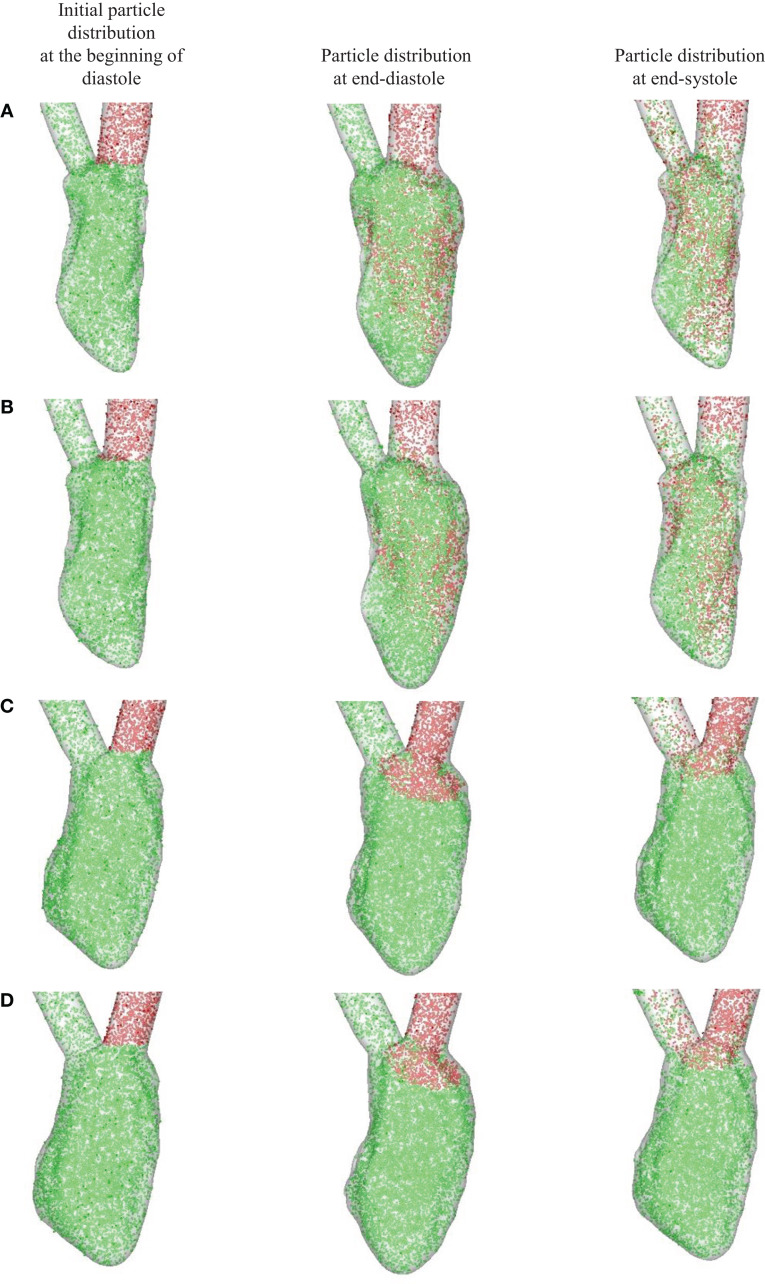
**Blood transport analyzed using Lagrangian particle tracking**. Red and green dots represent the atrial and ventricular blood cells, respectively. **(A)** Normal heart with SR activation; **(B)** normal heart with LBBB activation; **(C)** failing heart with SR activation; **(D)** failing heart with LBBB activation. SR, sinus rhythm; LBBB, left bundle branch block.

Figure 9 shows the Lagrangian blood transport in the LV; blood cells that enter the ventricle from the atrium during diastole are represented by red dots and blood cells located within the ventricle at the start of diastole by green dots. The number density (i.e., number of blood cells per unit volume) for both is assumed to be the same. For the normal hearts (Figures 9A,B), the fresh atrial inflow moves toward the apex during diastole and reach the vicinity of the apex by end-diastole. It is interesting to point out that even through the ventricular vortex only convects slightly more than half way into the ventricle (Figures 6 and 8), blood cells from the atrium are induced to penetrate deeper into the ventricle. By end-systole, the atrial and ventricular blood cells are found to be well-mixed and this is in line with the study of Seo and Mittal (2013b) for the normal human ventricles. For the failing hearts (Figures 9C,D), the atrial blood does not penetrate far into the ventricle, and therefore does not significantly displace the ventricular blood. During systole, this atrial bolus of A-wave (or atrial kick) that persists near the mitral annulus, is ejected out of the ventricle. Thus, the atrial and ventricular blood cells remain mostly unmixed throughout the entire cycle and the ventricular blood cells in the LV remain stagnant in the majority of the LV for failing hearts (Figures 9C,D).

**Figure 10:**
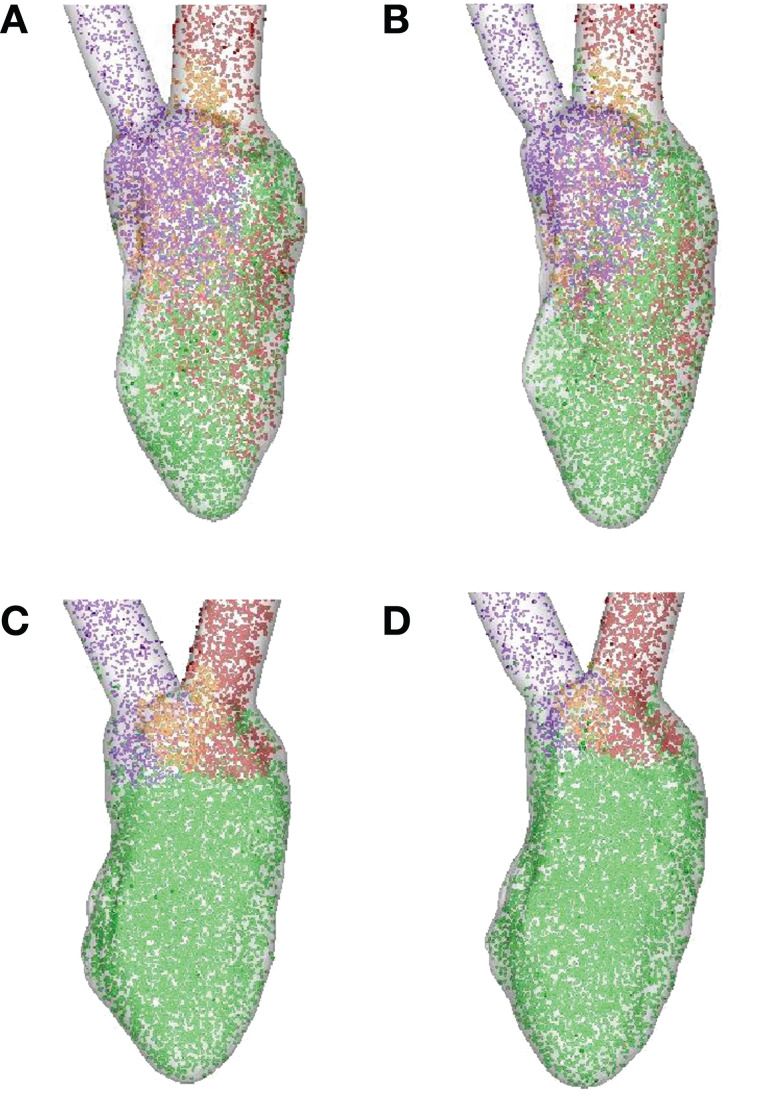
**Ventricular volume decomposition during a cardiac cycle (Bolger et al., 2007; Carlhall and Bolger, 2010; Hendabadi et al., 2013; Seo and Mittal, 2013b)**. The direct inflow or ejected atrial blood and delayed ejection or ejected ventricular blood cells from the previous cycle are represented in orange and purple, respectively, at the end-diastolic phase, while the red and green represent retained inflow and residual ventricular blood, respectively. **(A)** Normal heart with SR activation; **(B)** normal heart with LBBB activation; **(C)** failing heart with SR activation; **(D)** failing heart with LBBB activation. SR, sinus rhythm; LBBB, left bundle branch block.

In order to quantify the transport characteristics of the blood cells during the full cardiac cycle, we calculated the proportion of ejected atrial blood or DR and ejected ventricular blood or WR during the cardiac cycle (Bolger et al., 2007; Carlhall and Bolger, 2010; Hendabadi et al., 2013; Seo and Mittal, 2013b). Figure 10 shows the decomposed ventricular volume at the end-diastolic phase for all the four case studies. The direct inflow and delayed ejection blood volumes are represented using orange and purple colors, respectively, while the retained inflow and residual blood volumes are colored red and green, respectively. Further, the DR and the WR for all heart cases are presented in Table 2. Both DR and WR are found to be lower for the heart models with LBBB activation compared to the heart models with SR activation. This is not surprising given that the ejection fraction for LBBB activated heart models is lower compared to the heart models under normal SR (see Table 1). Likewise, the trends in WR also correspond to the trends in ejection fraction for all the heart models. However, it is interesting to note that although the DR for normal hearts is relatively lower than that of WR, although, this trend is reversed for the LBBB failing hearts. This indicates that for failing hearts the atrial blood forms a greater fraction of the ejected blood compared to the ventricular blood. Hence, the blood cells can remain in the ventricle for a long period of time with implications for ventricular thrombogenesis.

**Table 2:** **Comparison of metrics related to particle tracking for the four canine heart models presented in Table 1**.

	DR (%)	WR (%)	*N*_[1%]_
Normal heart	27.8	43.5	8
SR activation
Normal heart	22.1	36.6	10
LBBB activation
Failing heart	27.3	16.6	25
SR activation
Failing heart	17.0	8.0	55
LBBB activation

*SR, sinus rhythm; LBBB, left bundle branch block; DR, direct ejection ratio; WR, washout ratio; *N*_[1%]_, number of cycles required to reduce the fraction of residual ventricular blood to 1%*.

Washout ratio is associated with the fraction of ejected ventricular blood (Doenst et al., 2009; Seo and Mittal, 2013b) and can also be related to the residual blood ratio (RR) as (WR = 1 − RR) (Seo and Mittal, 2013b). This is a useful metric as one can now estimate the fraction of residual ventricular blood at the end of *N* cycles as (RR)*^N^* (Doenst et al., 2009; Seo and Mittal, 2013b). Therefore, the number of cycles required to reduce the ventricular blood constituents to 1% of initial ventricular blood volume can be computed as *N*_[1%]_ = −2/log_10_(RR) and is compared here for all the four cases in Table 2. We note that the *N*_[1%]_ for the normal canine heart estimated here is similar to that for the normal human heart (Seo and Mittal, 2013b). Second, it is noted that the washout efficiency deteriorates significantly for failing hearts, thereby increasing the residence time of blood cells in the ventricle.

### *P*–*V* loops

**Figure 11:**
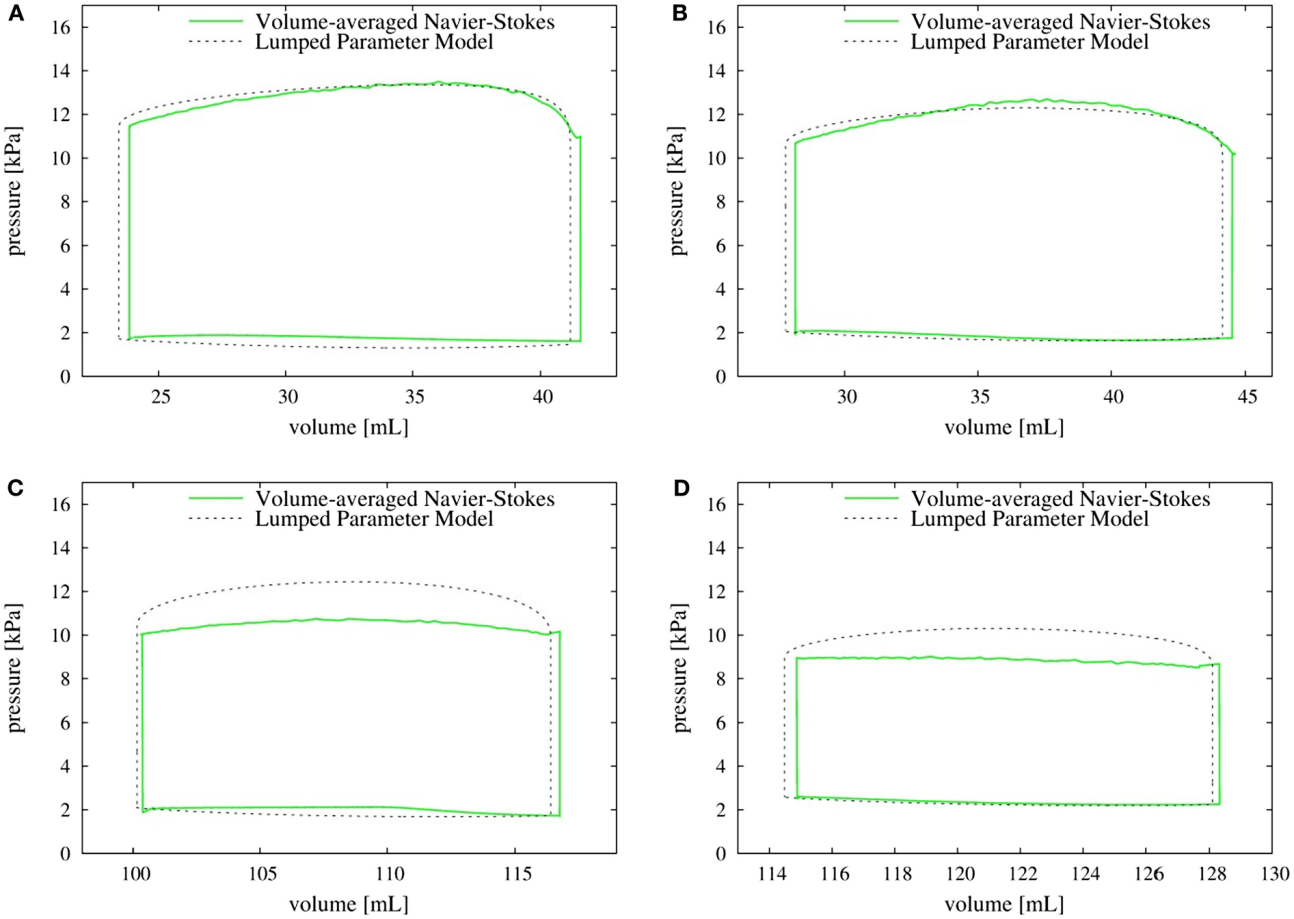
***P*–*V* loop: comparison of volume-averaged left ventricular pressure from the Navier–Stokes equations (solid line) with the pressure obtained from the lumped parameter model (dotted line)**. **(A)** Normal heart with SR activation; **(B)** normal heart with LBBB activation; **(C)** failing heart with SR activation; **(D)** failing heart with LBBB activation. SR, sinus rhythm; LBBB, left bundle branch block.

The left ventricular pressure–volume (*P*–*V*) loops for all the four canine hearts are plotted in Figure 11. The volume-averaged *P*–*V* loops are also compared with the corresponding loops obtained from the lumped parameter model (Kerckhoffs et al., 2007). The two *P*–*V* loops agree quite well for normal hearts with both the SR as well as the LBBB activation. By contrast, for the failing hearts, the lumped parameter model significantly over-predicts the systolic LV pressure when compared to the volume-averaged NSH model (10.7 versus 9.0 kPa for the SR model and 12.5 versus 10.3 kPa for the LBBB activation model). The models with LBBB activation (both normal and failing) generate lower systolic ventricular pressure compared to the ones with SR activation.

## Discussion

In this paper, we have described a methodology for multi-physics simulation of heart function by coupling a biophysically detailed multi-scale electromechanical model of the heart generated from DTMR data, with a high-fidelity NSH model. This one-way coupled model provides a unique capability to explore the complex effects of myocardial features and electromechanical abnormalities on LV hemodynamics.

Four canine heart models are simulated (a normal and a failing heart each under SR and with LBBB activation to simulate dyssynchrony) and the hemodynamics of these models are compared in order to demonstrate the usefulness of this novel methodology. During diastole, the failing hearts with lower ejection fraction have much smaller mitral inflow velocity than the normal hearts and ventricular blood is not efficiently washed out from these failing heart models. Hence, the blood cells can remain in the ventricle for a longer period of time (over 50 cardiac cycles), which leads to conditions conducive to ventricular thrombus formation (Richardson, 1973; Maze et al., 1989; Rayz et al., 2010). It is also known that stagnant intraventricular blood flow can cause low wall shear stress on the endocardium, which could also lead to more favorable conditions for thrombus formation (Richardson, 1973; Rayz et al., 2010). In our analysis, we noted that the apical region of the failing hearts is subjected to low velocities as well as low blood volume displacement, which is a marker for high risk of thrombus formation.

Accurate prediction of LV pressure is an essential component of most cardiac diagnostic procedures and we evaluate the prediction of typical LEM against the current higher-fidelity NSH model. While these predictions match well for the normal hearts, the lumped parameter model over-predicts the systolic pressure by up to about 20% for the failing hearts; however, the cause for this discrepancy is not clear. The simulations also indicate that the pressure is nearly uniform in the entire ventricle. The NSH simulations, therefore indicate that although the inherent approximation of uniform pressure in the LV for lumped-element models is reasonable, the LEMs need to be recalibrated for failing hearts.

It is also useful to point out the limitations of the current modeling approach. The current model is based on a one-way coupling between the electromechanics model and the hemodynamics model. This has advantages in that it allows the electromechanical and hemodynamic domains to be integrated in time with time steps that are most suitable for each domain. However, a two-way coupling would allow for feedback of the ventricular pressure from the hemodynamic solver back to the electromechanical model and improve the fidelity of the overall model. Furthermore, this model lacks the mitral and aortic valves in addition to assuming a smooth ventricle devoid of trabeculae and papillary muscles. The mitral valve and trabeculae in particular could have a significant effect on the ventricular hemodynamics (Seo et al., 2014; Vedula et al., 2015). However, since all the models explored here are similar in that respect, we expect that the comparative study still provides a reasonable estimation of the key trends that have been discussed here.

Finally, we point out that while the two components of the present coupled model (the combined CE and the Navier–Stokes hemodynamics) have been individually subjected to detailed verification and validation in separate studies (Constantino et al., 2013; Vedula et al., 2014), and that the heart-function model resulting from the coupling of the two does generate some qualitative features of the LV flow that match those from previous studies, a detailed validation of this method needs to be undertaken in the future. In the absence of such strong validation, we provide data regarding the canine LV kinematics in the online supplement, and this should enable other researchers to examine our model in detail and even assist in the replication of our results.

## Conflict of Interest Statement

The authors declare that the research was conducted in the absence of any commercial or financial relationships that could be construed as a potential conflict of interest.

## Supplementary Material

The Supplementary Material for this article can be found online at http://journal.frontiersin.org/article/10.3389/fbioe.2015.00140

## Footnotes

^1^http://www.sci.utah.edu/cibc-software/seg3d.html

^2^http://biomedical.materialise.com/mimics
