# Supplementary material for: A New MRI-Based Model of Heart Function with Coupled Hemodynamics and Application to Normal and Diseased Canine Left Ventricles
Source: Front Bioeng Biotechnol. 2015 Sep 23;3:140. doi: 10.3389/fbioe.2015.00140 (PMC4585083; doi:10.3389/fbioe.2015.00140)
Supplement: Supplementary file 1 [file Data_Sheet_1.PDF]

Supplementary Material

A new MRI-based model of heart function with coupled hemodynamics and application to normal and diseased canine left ventricles

Data Sheet 1

Young Joon Choi, Jason Constantino, Vijay Vedula, Natalia Trayanova, Rajat Mittal*

***Correspondence:** Corresponding Author: mittal@jhu.edu

1. Supplementary Data

1.1 Introduction

LV marker datasets are provided for four cases:

a. Normal LV beating under sinus rhythm (SR) conditions (Data Sheets 2&3)
b. Normal LV with dyssynchrony due to LBBB activation (Data Sheets 4&5)
c. Failing LV beating under sinus rhythm (SR) conditions (Data Sheets 6&7)
d. Failing LV with dyssynchrony due to LBBB activation (Data Sheets 8&9)

Datasets are provided for both diastole and systole and are presented in CSV (comma separated values) format.

1.2 Folder Structure

The data folder consists of eight subfolders named using the following structure:

Case<number>_<LV condition>_<beating condition>_<cycle phase>

where, "number" ranges from 1-4 for the cases described above; "LV condition" is either "Normal" or "HF" implying failing heart; "beating condition" which is either "SR" that stands for sinus rhythm or "LBBB" signifying dyssynchrony due to LBBB activation; and “cycle phase” is either diastole or systole.
e.g., Case3_Normal_LBBB_Systole

Within each of these subfolders, there exists two folders namely, “nodes” and “elems” each of which consists of nodal coordinates and element connectivity, respectively of the triangulated LV marker dataset.

1.3 File Naming Convention

Each of these subfolders contains LV marker data set for both diastole and systole written in CSV format. The naming convention template is,

case<number>_<cycle phase>_time_<time stamp>.csv

where, "number" ranges from 1-4 as described above; "cycle phase" is either "diastole" or "systole"; "time stamp" is the time instant during the cardiac cycle in seconds.
e.g., case2_diastole_time_0.1500.csv
